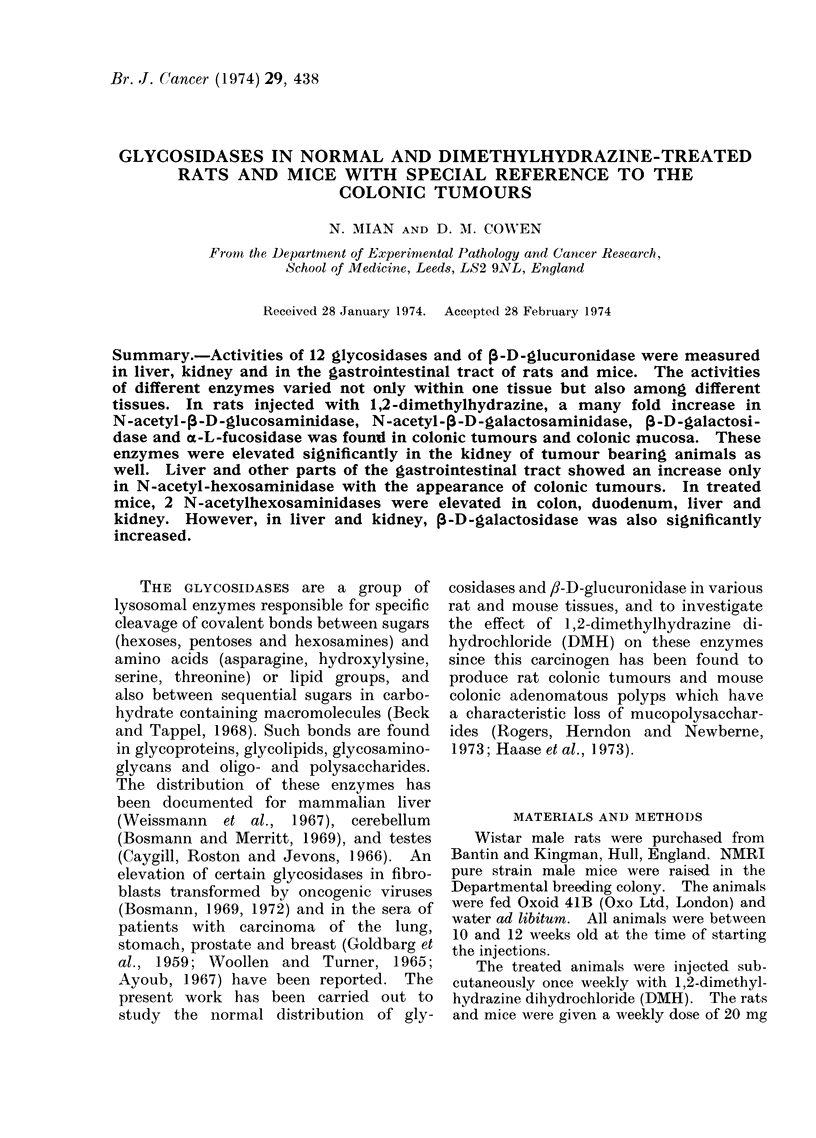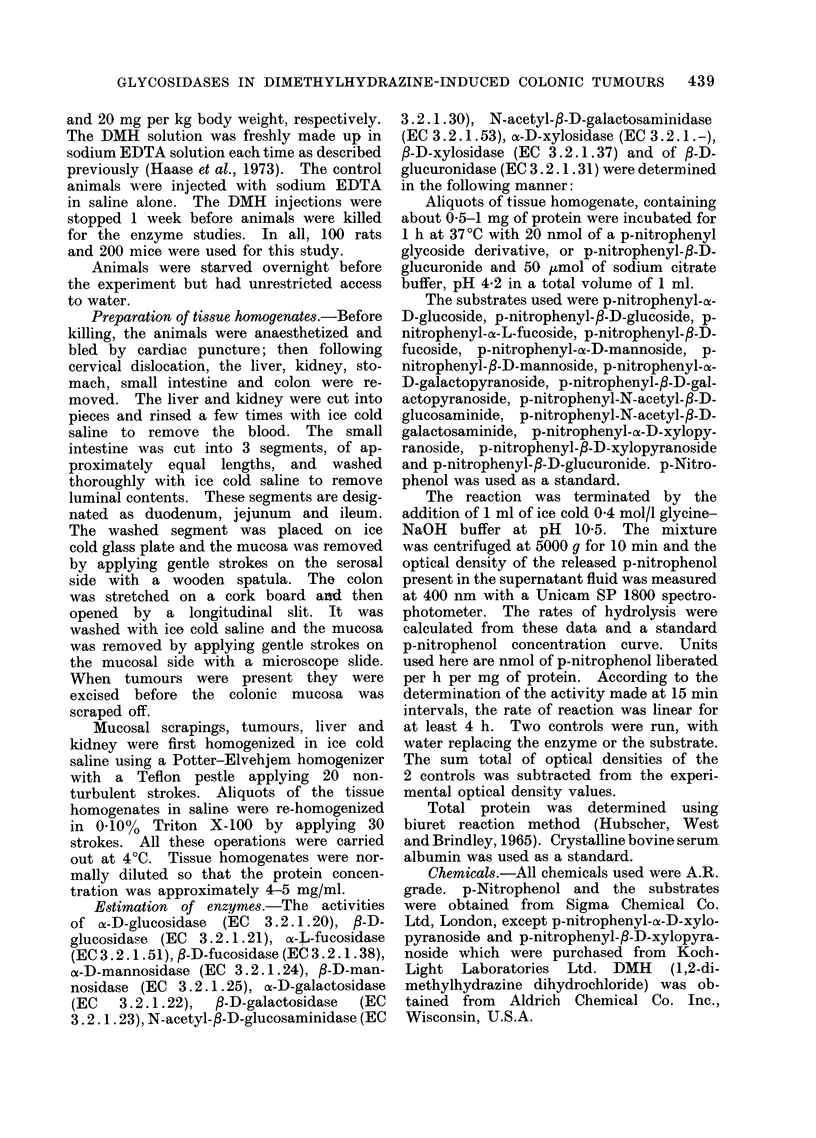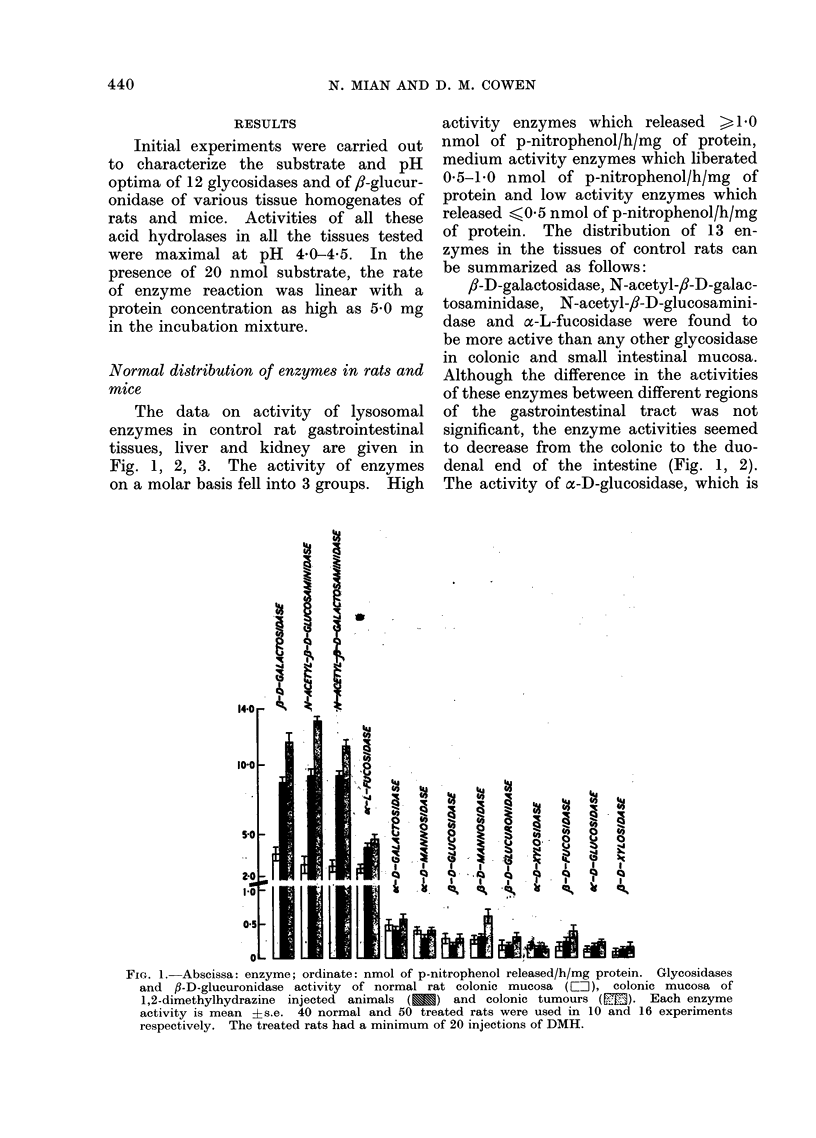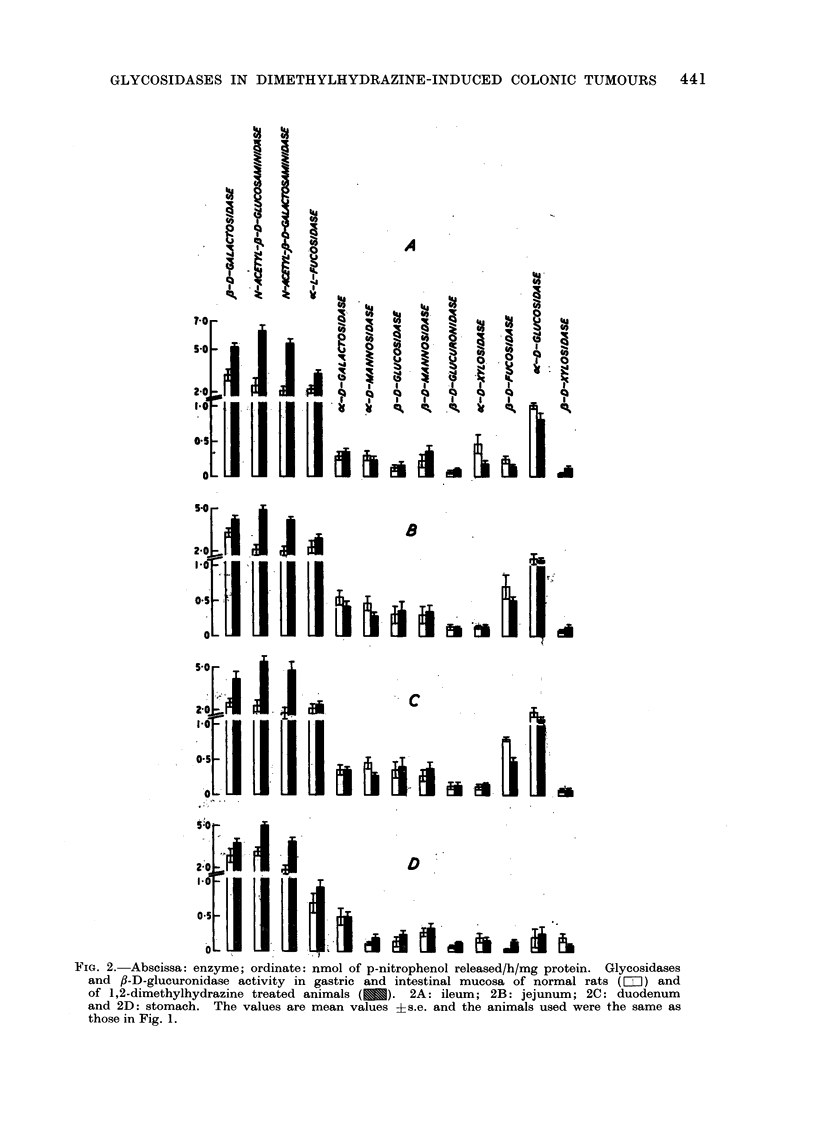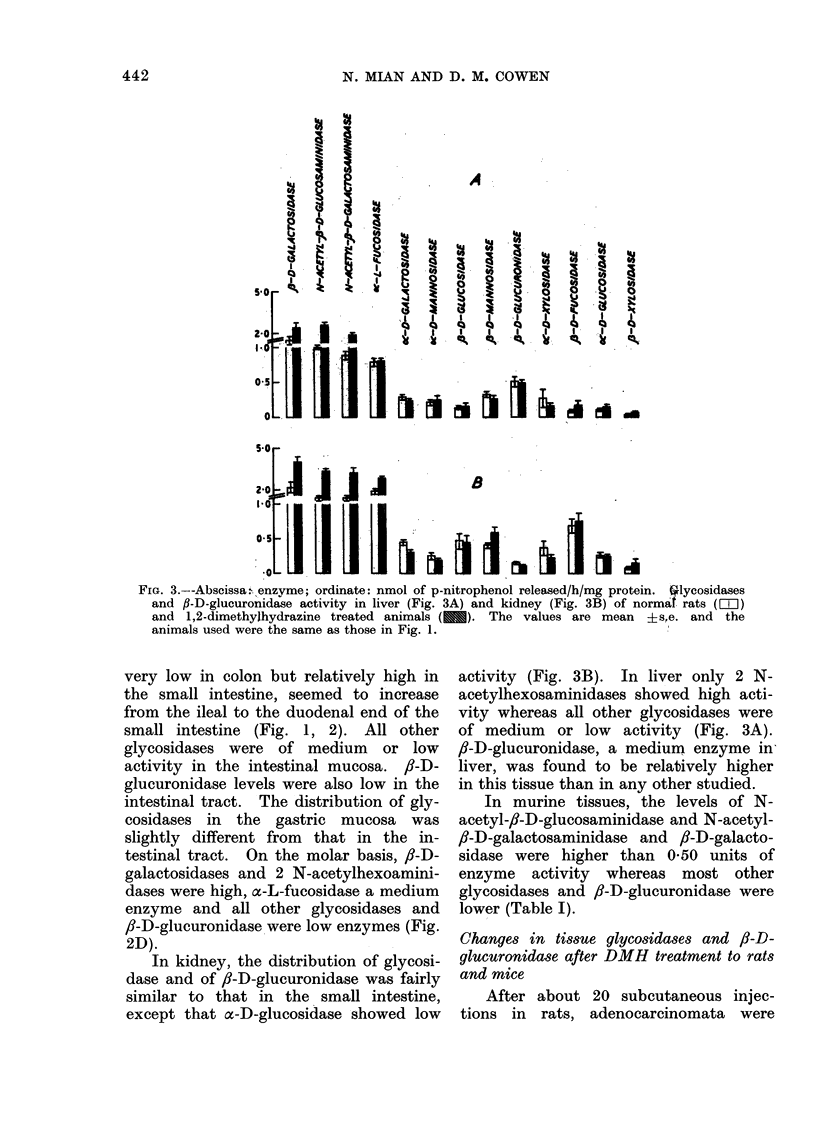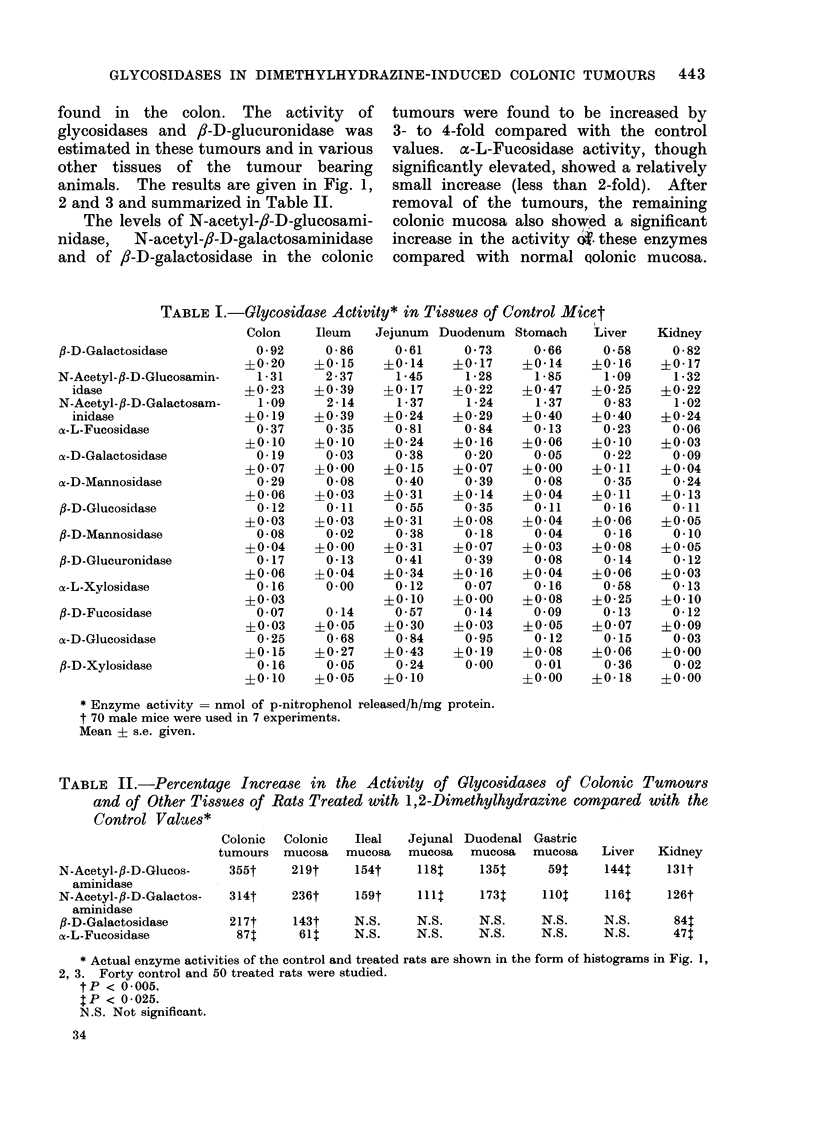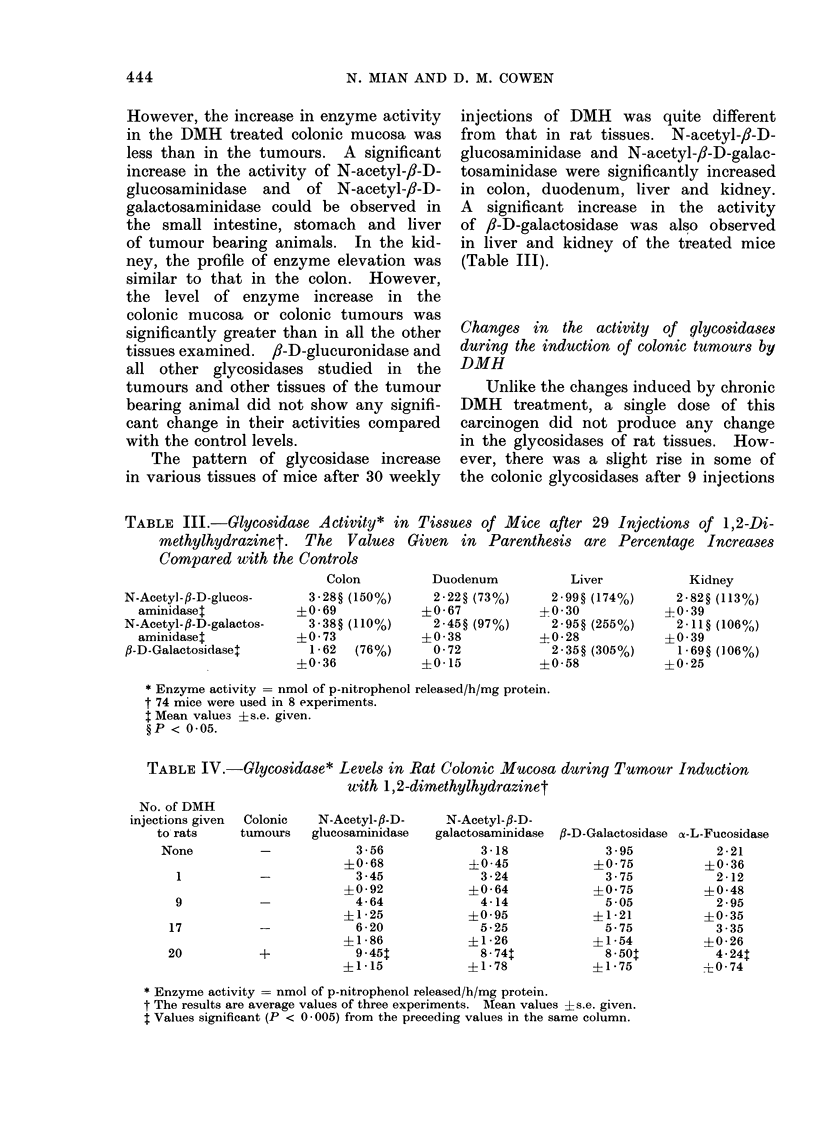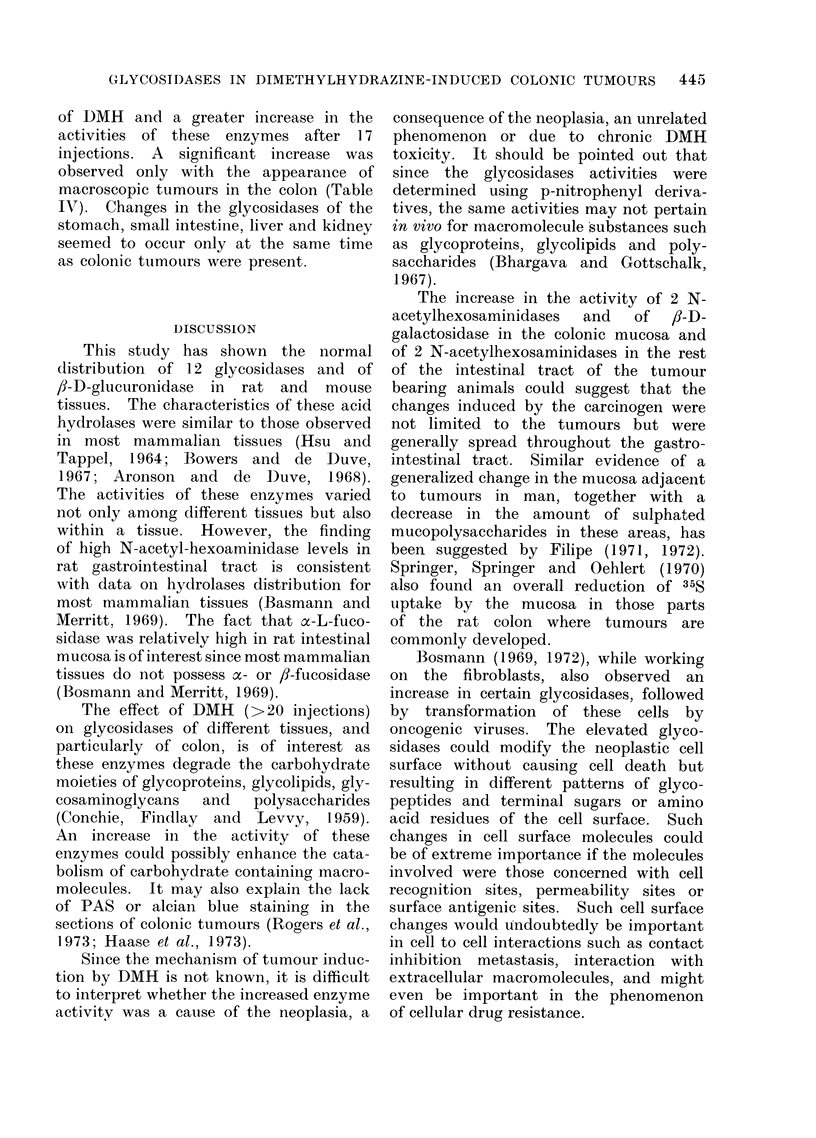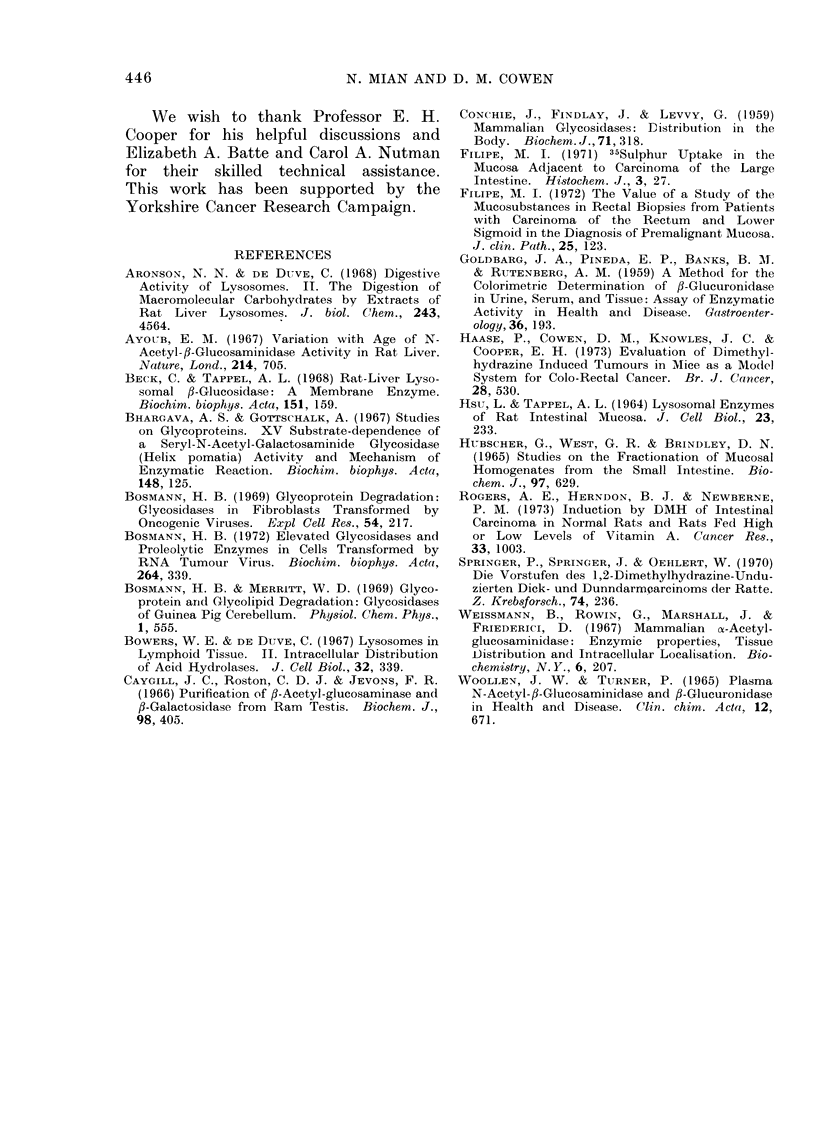# Glycosidases in Normal and Dimethylhydrazine-Treated Rats and Mice with Special Reference to the Colonic Tumours

**DOI:** 10.1038/bjc.1974.96

**Published:** 1974-06

**Authors:** N. Mian, D. M. Cowen

## Abstract

Activities of 12 glycosidases and of β-D-glucuronidase were measured in liver, kidney and in the gastrointestinal tract of rats and mice. The activities of different enzymes varied not only within one tissue but also among different tissues. In rats injected with 1,2-dimethylhydrazine, a many fold increase in N-acetyl-β-D-glucosaminidase, N-acetyl-β-D-galactosaminidase, β-D-galactosidase and α-L-fucosidase was found in colonic tumours and colonic mucosa. These enzymes were elevated significantly in the kidney of tumour bearing animals as well. Liver and other parts of the gastrointestinal tract showed an increase only in N-acetyl-hexosaminidase with the appearance of colonic tumours. In treated mice, 2 N-acetylhexosaminidases were elevated in colon, duodenum, liver and kidney. However, in liver and kidney, β-D-galactosidase was also significantly increased.


*Br. J. Cancer* (1974) **29**, 438

### GLYCOSIDASES IN NORMAL AND DIMETHYLHYDRAZINE-TREATED RATS AND MICE WITH SPECIAL REFERENCE TO THE COLONIC TUMOURS

N. MIAN AND D. M. COWEN

*From the Department of Experimental Pathology and Cancer Research, School of Medicine, Leeds, LS2 9NL, England*

Received 28 January 1974. Accepted 28 February 1974

**Summary.—Activities of 12 glycosidases and of β-D-glucuronidase were measured in liver, kidney and in the gastrointestinal tract of rats and mice. The activities of different enzymes varied not only within one tissue but also among different tissues. In rats injected with 1,2-dimethylhydrazine, a many fold increase in N-acetyl-β-D-glucosaminidase, N-acetyl-β-D-galactosaminidase, β-D-galactosidase and α-L-fucosidase was found in colonic tumours and colonic mucosa. These enzymes were elevated significantly in the kidney of tumour bearing animals as well. Liver and other parts of the gastrointestinal tract showed an increase only in N-acetyl-hexosaminidase with the appearance of colonic tumours. In treated mice, 2 N-acetylhexosaminidases were elevated in colon, duodenum, liver and kidney. However, in liver and kidney, β-D-galactosidase was also significantly increased.**

THE GLYCOSIDASES are a group of lysosomal enzymes responsible for specific cleavage of covalent bonds between sugars (hexoses, pentoses and hexosamines) and amino acids (asparagine, hydroxylysine, serine, threonine) or lipid groups, and also between sequential sugars in carbohydrate containing macromolecules (Beck and Tappel, 1968). Such bonds are found in glycoproteins, glycolipids, glycosaminoglycans and oligo- and polysaccharides. The distribution of these enzymes has been documented for mammalian liver (Weissmann *et al.*, 1967), cerebellum (Bosmann and Merritt, 1969), and testes (Caygill, Roston and Jevons, 1966). An elevation of certain glycosidases in fibroblasts transformed by oncogenic viruses (Bosmann, 1969, 1972) and in the sera of patients with carcinoma of the lung, stomach, prostate and breast (Goldbarg *et al.*, 1959; Woollen and Turner, 1965; Ayoub, 1967) have been reported. The present work has been carried out to study the normal distribution of glycosidases and β-D-glucuronidase in various rat and mouse tissues, and to investigate the effect of 1,2-dimethylhydrazine dihydrochloride (DMH) on these enzymes since this carcinogen has been found to produce rat colonic tumours and mouse colonic adenomatous polyps which have a characteristic loss of mucopolysaccharides (Rogers, Herndon and Newberne, 1973; Haase *et al.*, 1973).

### MATERIALS AND METHODS

Wistar male rats were purchased from Bantin and Kingman, Hull, England. NMRI pure strain male mice were raised in the Departmental breeding colony. The animals were fed Oxoid 41B (Oxo Ltd, London) and water *ad libitum*. All animals were between 10 and 12 weeks old at the time of starting the injections.

The treated animals were injected subcutaneously once weekly with 1,2-dimethylhydrazine dihydrochloride (DMH). The rats and mice were given a weekly dose of 20 mg and 20 mg per kg body weight, respectively. The DMH solution was freshly made up in sodium EDTA solution each time as described previously (Haase *et al.*, 1973). The control animals were injected with sodium EDTA in saline alone. The DMH injections were stopped 1 week before animals were killed for the enzyme studies. In all, 100 rats and 200 mice were used for this study.

Animals were starved overnight before the experiment but had unrestricted access to water.

*Preparation of tissue homogenates.*—Before killing, the animals were anaesthetized and bled by cardiac puncture; then following cervical dislocation, the liver, kidney, stomach, small intestine and colon were removed. The liver and kidney were cut into pieces and rinsed a few times with ice cold saline to remove the blood. The small intestine was cut into 3 segments, of approximately equal lengths, and washed thoroughly with ice cold saline to remove luminal contents. These segments are designated as duodenum, jejunum and ileum. The washed segment was placed on ice cold glass plate and the mucosa was removed by applying gentle strokes on the serosal side with a wooden spatula. The colon was stretched on a cork board and then opened by a longitudinal slit. It was washed with ice cold saline and the mucosa was removed by applying gentle strokes on the mucosal side with a microscope slide. When tumours were present they were excised before the colonic mucosa was scraped off.

Mucosal scrapings, tumours, liver and kidney were first homogenized in ice cold saline using a Potter–Elvehjem homogenizer with a Teflon pestle applying 20 non-turbulent strokes. Aliquots of the tissue homogenates in saline were re-homogenized in 0·10% Triton X-100 by applying 30 strokes. All these operations were carried out at 4°C. Tissue homogenates were normally diluted so that the protein concentration was approximately 4–5 mg/ml.

*Estimation of enzymes.*—The activities of α-D-glucosidase (EC 3.2.1.20), β-D-glucosidase (EC 3.2.1.21), α-L-fucosidase (EC 3.2.1.51), β-D-fucosidase (EC 3.2.1.38), α-D-mannosidase (EC 3.2.1.24), β-D-mannosidase (EC 3.2.1.25), α-D-galactosidase (EC 3.2.1.22), β-D-galactosidase (EC 3.2.1.23), N-acetyl-β-D-glucosaminidase (EC 3.2.1.30), N-acetyl-β-D-galactosaminidase (EC 3.2.1.53), α-D-xylosidase (EC 3.2.1.-), β-D-xylosidase (EC 3.2.1.37) and of β-D-glucuronidase (EC 3.2.1.31) were determined in the following manner:

Aliquots of tissue homogenate, containing about 0·5–1 mg of protein were incubated for 1 h at 37°C with 20 nmol of a p-nitrophenyl glycoside derivative, or p-nitrophenyl-β-D-glucuronide and 50 μmol of sodium citrate buffer, pH 4·2 in a total volume of 1 ml.

The substrates used were p-nitrophenyl-α-D-glucoside, p-nitrophenyl-β-D-glucoside, p-nitrophenyl-α-L-fucoside, p-nitrophenyl-β-D-fucoside, p-nitrophenyl-α-D-mannoside, p-nitrophenyl-β-D-mannoside, p-nitrophenyl-α-D-galactopyranoside, p-nitrophenyl-β-D-galactopyranoside, p-nitrophenyl-N-acetyl-β-D-glucosaminide, p-nitrophenyl-N-acetyl-β-D-galactosaminide, p-nitrophenyl-α-D-xylopyranoside, p-nitrophenyl-β-D-xylopyranoside and p-nitrophenyl-β-D-glucuronide. p-Nitrophenol was used as a standard.

The reaction was terminated by the addition of 1 ml of ice cold 0·4 mol/l glycine–NaOH buffer at pH 10·5. The mixture was centrifuged at 5000 *g* for 10 min and the optical density of the released p-nitrophenol present in the supernatant fluid was measured at 400 nm with a Unicam SP 1800 spectrophotometer. The rates of hydrolysis were calculated from these data and a standard p-nitrophenol concentration curve. Units used here are nmol of p-nitrophenol liberated per h per mg of protein. According to the determination of the activity made at 15 min intervals, the rate of reaction was linear for at least 4 h. Two controls were run, with water replacing the enzyme or the substrate. The sum total of optical densities of the 2 controls was subtracted from the experimental optical density values.

Total protein was determined using biuret reaction method (Hubscher, West and Brindley, 1965). Crystalline bovine serum albumin was used as a standard.

*Chemicals.*—All chemicals used were A.R. grade. p-Nitrophenol and the substrates were obtained from Sigma Chemical Co. Ltd, London, except p-nitrophenyl-α-D-xylopyranoside and p-nitrophenyl-β-D-xylopyranoside which were purchased from Koch-Light Laboratories Ltd. DMH (1,2-dimethylhydrazine dihydrochloride) was obtained from Aldrich Chemical Co. Inc., Wisconsin, U.S.A.

RESULTS

Initial experiments were carried out to characterize the substrate and pH optima of 12 glycosidases and of β-glucuronidase of various tissue homogenates of rats and mice. Activities of all these acid hydrolases in all the tissues tested were maximal at pH 4·0–4·5. In the presence of 20 nmol substrate, the rate of enzyme reaction was linear with a protein concentration as high as 5·0 mg in the incubation mixture.

*Normal distribution of enzymes in rats and mice*

The data on activity of lysosomal enzymes in control rat gastrointestinal tissues, liver and kidney are given in Fig. 1, 2, 3. The activity of enzymes on a molar basis fell into 3 groups. High activity enzymes which released $\geqslant 1{\cdot}0$ nmol of p-nitrophenol/h/mg of protein, medium activity enzymes which liberated 0·5–1·0 nmol of p-nitrophenol/h/mg of protein and low activity enzymes which released $\leqslant 0{\cdot}5$ nmol of p-nitrophenol/h/mg of protein. The distribution of 13 enzymes in the tissues of control rats can be summarized as follows:

β-D-galactosidase, N-acetyl-β-D-galactosaminidase, N-acetyl-β-D-glucosaminidase and α-L-fucosidase were found to be more active than any other glycosidase in colonic and small intestinal mucosa. Although the difference in the activities of these enzymes between different regions of the gastrointestinal tract was not significant, the enzyme activities seemed to decrease from the colonic to the duodenal end of the intestine (Fig. 1, 2). The activity of α-D-glucosidase, which is

FIG. 1.—Abscissa: enzyme; ordinate: nmol of p-nitrophenol released/h/mg protein. Glycosidases and β-D-glucuronidase activity of normal rat colonic mucosa (□), colonic mucosa of 1,2-dimethylhydrazine injected animals (▨) and colonic tumours (░). Each enzyme activity is mean ±s.e. 40 normal and 50 treated rats were used in 10 and 16 experiments respectively. The treated rats had a minimum of 20 injections of DMH.

FIG. 2.—Abscissa: enzyme; ordinate: nmol of p-nitrophenol released/h/mg protein. Glycosidases and β-D-glucuronidase activity in gastric and intestinal mucosa of normal rats (□) and of 1,2-dimethylhydrazine treated animals (▨). 2A: ileum; 2B: jejunum; 2C: duodenum and 2D: stomach. The values are mean values ±s.e. and the animals used were the same as those in Fig. 1.

FIG. 3.—Abscissa: enzyme; ordinate: nmol of p-nitrophenol released/h/mg protein. Glycosidases and β-D-glucuronidase activity in liver (Fig. 3A) and kidney (Fig. 3B) of normal rats (□) and 1,2-dimethylhydrazine treated animals (▨). The values are mean ±s.e. and the animals used were the same as those in Fig. 1.

very low in colon but relatively high in the small intestine, seemed to increase from the ileal to the duodenal end of the small intestine (Fig. 1, 2). All other glycosidases were of medium or low activity in the intestinal mucosa. β-D-glucuronidase levels were also low in the intestinal tract. The distribution of glycosidases in the gastric mucosa was slightly different from that in the intestinal tract. On the molar basis, β-D-galactosidases and 2 N-acetylhexoaminidases were high, α-L-fucosidase a medium enzyme and all other glycosidases and β-D-glucuronidase were low enzymes (Fig. 2D).

In kidney, the distribution of glycosidase and of β-D-glucuronidase was fairly similar to that in the small intestine, except that α-D-glucosidase showed low activity (Fig. 3B). In liver only 2 N-acetylhexosaminidases showed high activity whereas all other glycosidases were of medium or low activity (Fig. 3A). β-D-glucuronidase, a medium enzyme in liver, was found to be relatively higher in this tissue than in any other studied.

In murine tissues, the levels of N-acetyl-β-D-glucosaminidase and N-acetyl-β-D-galactosaminidase and β-D-galactosidase were higher than 0·50 units of enzyme activity whereas most other glycosidases and β-D-glucuronidase were lower (Table I).

*Changes in tissue glycosidases and β-D-glucuronidase after DMH treatment to rats and mice*

After about 20 subcutaneous injections in rats, adenocarcinomata were found in the colon. The activity of glycosidases and β-D-glucuronidase was estimated in these tumours and in various other tissues of the tumour bearing animals. The results are given in Fig. 1, 2 and 3 and summarized in Table II.

The levels of N-acetyl-β-D-glucosaminidase, N-acetyl-β-D-galactosaminidase and of β-D-galactosidase in the colonic tumours were found to be increased by 3- to 4-fold compared with the control values. α-L-Fucosidase activity, though significantly elevated, showed a relatively small increase (less than 2-fold). After removal of the tumours, the remaining colonic mucosa also showed a significant increase in the activity of these enzymes compared with normal colonic mucosa.

TABLE I.—*Glycosidase Activity* in Tissues of Control Mice†*

| | Colon | Ileum | Jejunum | Duodenum | Stomach | Liver | Kidney |
|---|---|---|---|---|---|---|---|
| β-D-Galactosidase | 0·92 | 0·86 | 0·61 | 0·73 | 0·66 | 0·58 | 0·82 |
| | ±0·20 | ±0·15 | ±0·14 | ±0·17 | ±0·14 | ±0·16 | ±0·17 |
| N-Acetyl-β-D-Glucosamin- | 1·31 | 2·37 | 1·45 | 1·28 | 1·85 | 1·09 | 1·32 |
| idase | ±0·23 | ±0·39 | ±0·17 | ±0·22 | ±0·47 | ±0·25 | ±0·22 |
| N-Acetyl-β-D-Galactosam- | 1·09 | 2·14 | 1·37 | 1·24 | 1·37 | 0·83 | 1·02 |
| inidase | ±0·19 | ±0·39 | ±0·24 | ±0·29 | ±0·40 | ±0·40 | ±0·24 |
| α-L-Fucosidase | 0·37 | 0·35 | 0·81 | 0·84 | 0·13 | 0·23 | 0·06 |
| | ±0·10 | ±0·10 | ±0·24 | ±0·16 | ±0·06 | ±0·10 | ±0·03 |
| α-D-Galactosidase | 0·19 | 0·03 | 0·38 | 0·20 | 0·05 | 0·22 | 0·09 |
| | ±0·07 | ±0·00 | ±0·15 | ±0·07 | ±0·00 | ±0·11 | ±0·04 |
| α-D-Mannosidase | 0·29 | 0·08 | 0·40 | 0·39 | 0·08 | 0·35 | 0·24 |
| | ±0·06 | ±0·03 | ±0·31 | ±0·14 | ±0·04 | ±0·11 | ±0·13 |
| β-D-Glucosidase | 0·12 | 0·11 | 0·55 | 0·35 | 0·11 | 0·16 | 0·11 |
| | ±0·03 | ±0·03 | ±0·31 | ±0·08 | ±0·04 | ±0·06 | ±0·05 |
| β-D-Mannosidase | 0·08 | 0·02 | 0·38 | 0·18 | 0·04 | 0·16 | 0·10 |
| | ±0·04 | ±0·00 | ±0·31 | ±0·07 | ±0·03 | ±0·08 | ±0·05 |
| β-D-Glucuronidase | 0·17 | 0·13 | 0·41 | 0·39 | 0·08 | 0·14 | 0·12 |
| | ±0·06 | ±0·04 | ±0·34 | ±0·16 | ±0·04 | ±0·06 | ±0·03 |
| α-L-Xylosidase | 0·16 | 0·00 | 0·12 | 0·07 | 0·16 | 0·58 | 0·13 |
| | ±0·03 | | ±0·10 | ±0·00 | ±0·08 | ±0·25 | ±0·10 |
| β-D-Fucosidase | 0·07 | 0·14 | 0·57 | 0·14 | 0·09 | 0·13 | 0·12 |
| | ±0·03 | ±0·05 | ±0·30 | ±0·03 | ±0·05 | ±0·07 | ±0·09 |
| α-D-Glucosidase | 0·25 | 0·68 | 0·84 | 0·95 | 0·12 | 0·15 | 0·03 |
| | ±0·15 | ±0·27 | ±0·43 | ±0·19 | ±0·08 | ±0·06 | ±0·00 |
| β-D-Xylosidase | 0·16 | 0·05 | 0·24 | 0·00 | 0·01 | 0·36 | 0·02 |
| | ±0·10 | ±0·05 | ±0·10 | | ±0·00 | ±0·18 | ±0·00 |

* Enzyme activity = nmol of p-nitrophenol released/h/mg protein.
† 70 male mice were used in 7 experiments.
Mean ± s.e. given.

TABLE II.—*Percentage Increase in the Activity of Glycosidases of Colonic Tumours and of Other Tissues of Rats Treated with* 1,2-*Dimethylhydrazine compared with the Control Values**

| | Colonic tumours | Colonic mucosa | Ileal mucosa | Jejunal mucosa | Duodenal mucosa | Gastric mucosa | Liver | Kidney |
|---|---|---|---|---|---|---|---|---|
| N-Acetyl-β-D-Glucos-aminidase | 355† | 219† | 154† | 118‡ | 135‡ | 59‡ | 144‡ | 131† |
| N-Acetyl-β-D-Galactos-aminidase | 314† | 236† | 159† | 111‡ | 173‡ | 110‡ | 116‡ | 126† |
| β-D-Galactosidase | 217† | 143† | N.S. | N.S. | N.S. | N.S. | N.S. | 84‡ |
| α-L-Fucosidase | 87‡ | 61‡ | N.S. | N.S. | N.S. | N.S. | N.S. | 47‡ |

* Actual enzyme activities of the control and treated rats are shown in the form of histograms in Fig. 1, 2, 3. Forty control and 50 treated rats were studied.
† $P < 0{\cdot}005$.
‡ $P < 0{\cdot}025$.
N.S. Not significant.

However, the increase in enzyme activity in the DMH treated colonic mucosa was less than in the tumours. A significant increase in the activity of N-acetyl-β-D-glucosaminidase and of N-acetyl-β-D-galactosaminidase could be observed in the small intestine, stomach and liver of tumour bearing animals. In the kidney, the profile of enzyme elevation was similar to that in the colon. However, the level of enzyme increase in the colonic mucosa or colonic tumours was significantly greater than in all the other tissues examined. β-D-glucuronidase and all other glycosidases studied in the tumours and other tissues of the tumour bearing animal did not show any significant change in their activities compared with the control levels.

The pattern of glycosidase increase in various tissues of mice after 30 weekly injections of DMH was quite different from that in rat tissues. N-acetyl-β-D-glucosaminidase and N-acetyl-β-D-galactosaminidase were significantly increased in colon, duodenum, liver and kidney. A significant increase in the activity of β-D-galactosidase was also observed in liver and kidney of the treated mice (Table III).

*Changes in the activity of glycosidases during the induction of colonic tumours by DMH*

Unlike the changes induced by chronic DMH treatment, a single dose of this carcinogen did not produce any change in the glycosidases of rat tissues. However, there was a slight rise in some of the colonic glycosidases after 9 injections

TABLE III.—*Glycosidase Activity* in Tissues of Mice after 29 Injections of 1,2-Dimethylhydrazine†. The Values Given in Parenthesis are Percentage Increases Compared with the Controls*

| | Colon | Duodenum | Liver | Kidney |
|---|---|---|---|---|
| N-Acetyl-β-D-glucosaminidase‡ | 3·28§ (150%) ±0·69 | 2·22§ (73%) ±0·67 | 2·99§ (174%) ±0·30 | 2·82§ (113%) ±0·39 |
| N-Acetyl-β-D-galactosaminidase‡ | 3·38§ (110%) ±0·73 | 2·45§ (97%) ±0·38 | 2·95§ (255%) ±0·28 | 2·11§ (106%) ±0·39 |
| β-D-Galactosidase‡ | 1·62 (76%) ±0·36 | 0·72 ±0·15 | 2·35§ (305%) ±0·58 | 1·69§ (106%) ±0·25 |

* Enzyme activity = nmol of p-nitrophenol released/h/mg protein.
† 74 mice were used in 8 experiments.
‡ Mean values ±s.e. given.
§ $P < 0{\cdot}05$.

TABLE IV.—*Glycosidase* Levels in Rat Colonic Mucosa during Tumour Induction with 1,2-dimethylhydrazine†*

| No. of DMH injections given to rats | Colonic tumours | N-Acetyl-β-D-glucosaminidase | N-Acetyl-β-D-galactosaminidase | β-D-Galactosidase | α-L-Fucosidase |
|---|---|---|---|---|---|
| None | — | 3·56 ±0·68 | 3·18 ±0·45 | 3·95 ±0·75 | 2·21 ±0·36 |
| 1 | — | 3·45 ±0·92 | 3·24 ±0·64 | 3·75 ±0·75 | 2·12 ±0·48 |
| 9 | — | 4·64 ±1·25 | 4·14 ±0·95 | 5·05 ±1·21 | 2·95 ±0·35 |
| 17 | — | 6·20 ±1·86 | 5·25 ±1·26 | 5·75 ±1·54 | 3·35 ±0·26 |
| 20 | + | 9·45‡ ±1·15 | 8·74‡ ±1·78 | 8·50‡ ±1·75 | 4·24‡ ±0·74 |

* Enzyme activity = nmol of p-nitrophenol released/h/mg protein.
† The results are average values of three experiments. Mean values ±s.e. given.
‡ Values significant ($P < 0{\cdot}005$) from the preceding values in the same column.

of DMH and a greater increase in the activities of these enzymes after 17 injections. A significant increase was observed only with the appearance of macroscopic tumours in the colon (Table IV). Changes in the glycosidases of the stomach, small intestine, liver and kidney seemed to occur only at the same time as colonic tumours were present.

### DISCUSSION

This study has shown the normal distribution of 12 glycosidases and of $\beta$-D-glucuronidase in rat and mouse tissues. The characteristics of these acid hydrolases were similar to those observed in most mammalian tissues (Hsu and Tappel, 1964; Bowers and de Duve, 1967; Aronson and de Duve, 1968). The activities of these enzymes varied not only among different tissues but also within a tissue. However, the finding of high N-acetyl-hexoaminidase levels in rat gastrointestinal tract is consistent with data on hydrolases distribution for most mammalian tissues (Basmann and Merritt, 1969). The fact that $\alpha$-L-fucosidase was relatively high in rat intestinal mucosa is of interest since most mammalian tissues do not possess $\alpha$- or $\beta$-fucosidase (Bosmann and Merritt, 1969).

The effect of DMH ($>$20 injections) on glycosidases of different tissues, and particularly of colon, is of interest as these enzymes degrade the carbohydrate moieties of glycoproteins, glycolipids, glycosaminoglycans and polysaccharides (Conchie, Findlay and Levvy, 1959). An increase in the activity of these enzymes could possibly enhance the catabolism of carbohydrate containing macromolecules. It may also explain the lack of PAS or alcian blue staining in the sections of colonic tumours (Rogers *et al.*, 1973; Haase *et al.*, 1973).

Since the mechanism of tumour induction by DMH is not known, it is difficult to interpret whether the increased enzyme activity was a cause of the neoplasia, a consequence of the neoplasia, an unrelated phenomenon or due to chronic DMH toxicity. It should be pointed out that since the glycosidases activities were determined using p-nitrophenyl derivatives, the same activities may not pertain *in vivo* for macromolecule substances such as glycoproteins, glycolipids and polysaccharides (Bhargava and Gottschalk, 1967).

The increase in the activity of 2 N-acetylhexosaminidases and of $\beta$-D-galactosidase in the colonic mucosa and of 2 N-acetylhexosaminidases in the rest of the intestinal tract of the tumour bearing animals could suggest that the changes induced by the carcinogen were not limited to the tumours but were generally spread throughout the gastrointestinal tract. Similar evidence of a generalized change in the mucosa adjacent to tumours in man, together with a decrease in the amount of sulphated mucopolysaccharides in these areas, has been suggested by Filipe (1971, 1972). Springer, Springer and Oehlert (1970) also found an overall reduction of $^{35}S$ uptake by the mucosa in those parts of the rat colon where tumours are commonly developed.

Bosmann (1969, 1972), while working on the fibroblasts, also observed an increase in certain glycosidases, followed by transformation of these cells by oncogenic viruses. The elevated glycosidases could modify the neoplastic cell surface without causing cell death but resulting in different patterns of glycopeptides and terminal sugars or amino acid residues of the cell surface. Such changes in cell surface molecules could be of extreme importance if the molecules involved were those concerned with cell recognition sites, permeability sites or surface antigenic sites. Such cell surface changes would undoubtedly be important in cell to cell interactions such as contact inhibition metastasis, interaction with extracellular macromolecules, and might even be important in the phenomenon of cellular drug resistance.

We wish to thank Professor E. H. Cooper for his helpful discussions and Elizabeth A. Batte and Carol A. Nutman for their skilled technical assistance. This work has been supported by the Yorkshire Cancer Research Campaign.

### REFERENCES

ARONSON, N. N. & DE DUVE, C. (1968) Digestive Activity of Lysosomes. II. The Digestion of Macromolecular Carbohydrates by Extracts of Rat Liver Lysosomes. *J. biol. Chem.*, **243**, 4564.

AYOUB, E. M. (1967) Variation with Age of N-Acetyl-$\beta$-Glucosaminidase Activity in Rat Liver. *Nature, Lond.*, **214**, 705.

BECK, C. & TAPPEL, A. L. (1968) Rat-Liver Lysosomal $\beta$-Glucosidase: A Membrane Enzyme. *Biochim. biophys. Acta*, **151**, 159.

BHARGAVA, A. S. & GOTTSCHALK, A. (1967) Studies on Glycoproteins. XV Substrate-dependence of a Seryl-N-Acetyl-Galactosaminide Glycosidase (Helix pomatia) Activity and Mechanism of Enzymatic Reaction. *Biochim. biophys. Acta*, **148**, 125.

BOSMANN, H. B. (1969) Glycoprotein Degradation: Glycosidases in Fibroblasts Transformed by Oncogenic Viruses. *Expl Cell Res.*, **54**, 217.

BOSMANN, H. B. (1972) Elevated Glycosidases and Proleolytic Enzymes in Cells Transformed by RNA Tumour Virus. *Biochim. biophys. Acta*, **264**, 339.

BOSMANN, H. B. & MERRITT, W. D. (1969) Glycoprotein and Glycolipid Degradation: Glycosidases of Guinea Pig Cerebellum. *Physiol. Chem. Phys.*, **1**, 555.

BOWERS, W. E. & DE DUVE, C. (1967) Lysosomes in Lymphoid Tissue. II. Intracellular Distribution of Acid Hydrolases. *J. Cell Biol.*, **32**, 339.

CAYGILL, J. C., Roston, C. D. J. & JEVONS, F. R. (1966) Purification of $\beta$-Acetyl-glucosaminase and $\beta$-Galactosidase from Ram Testis. *Biochem. J.*, **98**, 405.

CONCHIE, J., FINDLAY, J. & LEVVY, G. (1959) Mammalian Glycosidases: Distribution in the Body. *Biochem. J.*, **71**, 318.

FILIPE, M. I. (1971) $^{35}$Sulphur Uptake in the Mucosa Adjacent to Carcinoma of the Large Intestine. *Histochem. J.*, **3**, 27.

FILIPE, M. I. (1972) The Value of a Study of the Mucosubstances in Rectal Biopsies from Patients with Carcinoma of the Rectum and Lower Sigmoid in the Diagnosis of Premalignant Mucosa. *J. clin. Path.*, **25**, 123.

GOLDBARG, J. A., PINEDA, E. P., BANKS, B. M. & RUTENBERG, A. M. (1959) A Method for the Colorimetric Determination of $\beta$-Glucuronidase in Urine, Serum, and Tissue: Assay of Enzymatic Activity in Health and Disease. *Gastroenterology*, **36**, 193.

HAASE, P., COWEN, D. M., KNOWLES, J. C. & COOPER, E. H. (1973) Evaluation of Dimethylhydrazine Induced Tumours in Mice as a Model System for Colo-Rectal Cancer. *Br. J. Cancer*, **28**, 530.

HSU, L. & TAPPEL, A. L. (1964) Lysosomal Enzymes of Rat Intestinal Mucosa. *J. Cell Biol.*, **23**, 233.

HUBSCHER, G., WEST, G. R. & BRINDLEY, D. N. (1965) Studies on the Fractionation of Mucosal Homogenates from the Small Intestine. *Biochem. J.*, **97**, 629.

ROGERS, A. E., HERNDON, B. J. & NEWBERNE, P. M. (1973) Induction by DMH of Intestinal Carcinoma in Normal Rats and Rats Fed High or Low Levels of Vitamin A. *Cancer Res.*, **33**, 1003.

SPRINGER, P., SPRINGER, J. & OEHLERT, W. (1970) Die Vorstufen des 1,2-Dimethylhydrazine-Unduzierten Dick- und Dunndarmparcinoms der Ratte. *Z. Krebsforsch.*, **74**, 236.

WEISSMANN, B., ROWIN, G., MARSHALL, J. & FRIEDERICI, D. (1967) Mammalian $\alpha$-Acetylglucosaminidase: Enzymic properties, Tissue Distribution and Intracellular Localisation. *Biochemistry, N.Y.*, **6**, 207.

WOOLLEN, J. W. & TURNER, P. (1965) Plasma N-Acetyl-$\beta$-Glucosaminidase and $\beta$-Glucuronidase in Health and Disease. *Clin. chim. Acta*, **12**, 671.